# Supplementary material for: The Role of a Cytokinin Antagonist in the Progression of Clubroot Disease
Source: Biomolecules. 2023 Feb 5;13(2):299. doi: 10.3390/biom13020299 (PMC9953476; doi:10.3390/biom13020299)
Supplement: Supplementary file 1 [file biomolecules-13-00299-s001.zip › biomolecules-2156171-supplementary.pdf]

# The Role of a Cytokinin Antagonist in the Progression of Clubroot Disease

**Jana Bíbová[1], Veronika Kábrtová[1], Veronika Večeřová[1], Zuzana Kučerová[2], Martin Hudeček[1], Lenka Plačková[1], Ondřej Novák[1], Miroslav Strnad[1,*] and Ondřej Plíhal[1,*]**

1 Laboratory of Growth Regulators, Faculty of Science, Institute of Experimental Botany of the Czech Academy of Sciences, Palacký University, Šlechtitelů 27, CZ-78371 Olomouc, Czech Republic

2 Department of Biophysics, Faculty of Science of Palacký University, Šlechtitelů 27, CZ-78371 Olomouc, Czech Republic

* Correspondence: miroslav.strnad@upol.cz (MS); ondrej.plihal@upol.cz (OP)

**Supplementary Information:**

**Supplementary Methods**

**Supplementary Methods S1.** Chemical synthesis and activity testing.

For the preparation of 6-(2-hydroxy-3-methylbenzylamino)purine (PI-55), 6-chloropurine was heated with 2-hydroxy-3-methylbenzylamine (to 90°C; 4 hours) in n-butanol with excess triethylamine. After lowering the reaction temperature, the precipitated product was filtered and rinsed with cold water and n-butanol and subsequently, it was crystalized from dimethylformamide. The melting points were determined on a Boetius stage and presented values are uncorrected. Analytical thin layer chromatography (TLC) was carried out using silica gel 60 WF254 plates (Merck). An EA1108 CHN analyzer (Fisons Instruments) was used for elemental analyses (C, H and N). ES+ mass spectra were recorded using a Waters ZMD 2000 mass spectrometer equipped with a direct probe, and a mass monitoring interval of 10 - 1500 amu. The spectra were collected using 3.0 second cyclical scans, applying a sample cone voltage of 25 V at a source block temperature of 150°C, desolvation temperature of 80°C and desolvation gas flow rate of 200 l/hour. The mass spectrometer was directly coupled to a MassLynx data system. NMR spectra were acquired in a Bruker Avance AV 300 spectrometer operating at a temperature of 300 K and a frequency of 300.13 MHz ($^{1}H$) or 75.48 MHz ($^{13}C$). Samples were prepared by dissolving the compounds in DMSO-d6. Tetramethylsilane (TMS) was used as the internal standard.

Elemental analysis (C, H, N) (Found: C, 60.85 ; H, 5.38 ; N, 27.29%. C13H13N5O calc.: C, 61.17; H, 5.13; N, 27.43%); TLC (chloroform : methanol : ammonia (8:2:0.1, v/v/v) as the mobile phase): single spot; m.p. 278.4-279.5°C; ES+ MS: m/z 255 $[MH]^{+}$; $^{1}H$-NMR (DMSO-d6, 300MHz): 13.03s (1H, N9H), 10.70s (1H, O1H), 8.45s (1H, N6H), 8.28s (1H, C8H), 8.16s (1H, C2H), 7.09d (1H, C13H), 7.00d (1H, C15H), 6.69tr (1H, C14H), 4.54s (2H, C9H), 2.16s (3H, C16H)

$^{13}C$-NMR (DMSO-d6, 75MHz): 153.94 (12C), 152.80 (6C), 152.00 (2C), 150.01 (4C), 140.01 (8C), 130.26 (15C), 128.63 (14C),126.13 (10C), 125.94 (11C), 119.52 (13C), 119.10 (5C), 40.44 (9C), 16.93 (16C).

Standard bioassays based on stimulation of cytokinin-dependent tobacco callus growth, the retention of chlorophyll in excised wheat leaves and the dark induction of betacyanin synthesis in Amaranthus cotyledons were carried out as described previously (Holub et al. 1998). Specifically, the relative activity of the prepared derivative at optimal concentration was compared with the activity of 6-benzylaminopurine (BAP), ($10^{-6}$ M BAP for the tobacco callus bioassay, $10^{-5}$ M BAP for the Amaranthus betacyanin bioassay, and $10^{-4}$ M BAP in the case of the senescence bioassay).

Holub J, Hanuš J, Hanke DE, Strnad M (1998) Biological activity of cytokinines derived from *Ortho*- and *Meta*-hydroxybenzyladenine. Plant Growth Regul 26: 109-115.

**Supplementary Table S1.** Primers for qRT-PCR.

| Gene | AGI No. | Primers 5´ - 3´ |
|---|---|---|
| **Housekeeping genes (Endogenous controls)** | | |
| *Act2* | *At3g18780* | TGGTCGTACAACCGGTATTGTG |
| | | ATCAGTAAGGTCACGTCCAGCAA |
| *Act7* | *At5g09810* | CTAGAGACAGCCAAGAGCAGTTC |
| | | GTTTCATGGATTCCAGGAGCTTC |
| *EF1α* | *At5g60390* | TGAGCACGCTCTTCTTGCTTTCA |
| | | GGTGGTGGCATCCATCTTGTTACA |
| ***MAPK/PTI marker genes*** | | |
| *ARR4* | *At1g10470* | GGTGGTATCGGAGGAATTGA |
| | | ACGCCATCCACTATCTACCG |
| *ARR5* | *At3g48100* | TGCCTGGGATGACTGGATATG |
| | | CTCCTTCTTCAAGACATCTATCG |
| *ARR7* | *At1g19050* | TACTCAATGCCAGGACTTTCAGG |
| | | TCTTTGAGACATTCTTGTATACGAGG |
| *ARR8* | *At2g41310* | AGTAGATGACAACGACCCAAAT |
| | | TATACTTCTAAACGCCGCTGAT |
| *ARR9* | *At3g57040* | ATG GGT ATG GCA GCA GAA TC |
| | | AAG CCT TTG AGC CAG AAT CA |
| *ARR16* | *At2g40670* | CGTAAACTCGTTGAGAGGTTGCTC |
| | | GCATTCTCTGCTGTTGTCACTTTG |
| *CKX1* | *At2g41510* | TAAGGGGAAGCAACCATATGTT |
| | | AACGGTCAAATGAAGGTAGTCT |
| *CKX4* | *At4g29740* | TCTTCTAAACCAAACCTCAACTTCTG |
| | | TGCGGTTGTTCCATTTGTTTC |
| *CKX5* | *At1g75450* | GGCGTTTTCAAGGGCATTTT |
| | | TCGTCCCATTTGTCTTTGTTCA |
| *CKX6* | *At3g63440* | GGGTACATGTGTCTGAGGTAAA |
| | | ATTTGTTCACTGGGTAGACGAT |
| *IPT1* | *At1g68460* | GAATCGGAAAGACAAAGTCGTC |
| | | ATGATCTCTGAAGGGAAACGAG |
| *IPT3* | *At3g63110* | CTCTCCATTGAATCCGTCCTAA |
| | | TCTTGTCAACTCTCTCAGACAC |
| *IPT5* | *At5g19040* | GACACTTACGAAGATTTCACGG |
| | | GCCTCGATGTAAGAATTGGAAC |
| *IPT7* | *At3g23630* | CAAGCAATCTCGAAACTCTCAG |
| | | AGACAAAGGAGTTAAGTACGGG |
| *LOG1* | *At2g28305* | GGGTTTGATTTCTCAAGCTGTT |
| | | CATCAGAGTGCTTAGCCATTTC |
| *LOG6* | *At5g03270* | GGTTTAATGGGTTTGGTGTCTC |
| | | CATCAGAATGCTTAGCCATCAC |

**Supplementary Table S2.**

Down and upregulated genes related to cytokinin signaling and metabolism. Expression changes were followed by qPCR and expressed as fold changes between mock-treated (0.1% DMSO) controls and infected/non-infected Col-0 or *ahk2 ahk3* double mutant with or without PI-55 treatment at a concentration of 10 µmol $l^{-1}$. All data were obtained 28 days after infection (dai) of control (ctrl) or clubroot-infected (inf) plants in three independent biological replicates.

| AGI No. | Description | Fold Change | | | | | |
|---|---|---|---|---|---|---|---|
| | | Col-0 ctrl. PI-55 | Col-0 inf. mock | Col-0 inf. PI-55 | *ahk2ahk3* ctrl. PI-55 | *ahk2ahk3* inf. mock | *ahk2ahk3* inf. PI-55 |
| AT1G10470 | Response regulator 4 (*ARR4*) | 1.05 | 1.61 | 1.48 | 1.58 | 0.90 | 1.12 |
| AT3G48100 | Response regulator 5 (*ARR5*) | 1.18 | 1.40 | 2.03 | 0.35 | 0.41 | 1.11 |
| AT1G19050 | Response regulator 7 (*ARR7*) | 1.22 | 1.10 | 2.08 | 1.64 | 0.68 | 1.92 |
| AT2G41310 | Response regulator 8 (*ARR8*) | 0.57 | 1.27 | 0.77 | 0.80 | 0.65 | 0.41 |
| AT3G57040 | Response regulator 9 (*ARR9*) | 0.56 | 1.20 | 0.78 | 0.56 | 0.58 | 0.62 |
| AT2G40670 | Response regulator 16 (*ARR16*) | 1.04 | 1.62 | 0.47 | 0.99 | 3.64 | 0.75 |
| AT2G41510 | Cytokinin oxidase/dehydrogenase 1 (*CKX1*) | 0.72 | 0.88 | 0.41 | 1.20 | 0.48 | 1.07 |
| AT4G29740 | Cytokinin oxidase/dehydrogenase 4 (*CKX4*) | 1.67 | 2.42 | 5.14 | 0.54 | 0.31 | 1.63 |
| AT1G75450 | Cytokinin oxidase/dehydrogenase 5 (*CKX5*) | 1.22 | 0.85 | 1.64 | 0.95 | 0.91 | 0.77 |
| AT3G63440 | Cytokinin oxidase/dehydrogenase 6 (*CKX6*) | 1.04 | 0.88 | 1.14 | 0.32 | 0.32 | 0.40 |
| AT1G68460 | Isopentenyltransferase 1 (*IPT1*) | 0.88 | 0.78 | 1.02 | 0.78 | 1.36 | 1.75 |
| AT3G63110 | Isopentenyltransferase 3 (*IPT3*) | 0.80 | 1.10 | 0.49 | 0.74 | 0.90 | 0.70 |
| AT5G19040 | Isopentenyltransferase 5 (*IPT5*) | 0.44 | 1.27 | 0.65 | 0.63 | 0.82 | 0.94 |
| AT3G23630 | Isopentenyltransferase 7 (*IPT7*) | 0.61 | 0.95 | 0.96 | 1.02 | 1.09 | 1.39 |
| AT2G28305 | LONELY GUY 1 (*LOG1*) | 1.03 | 0.91 | 1.37 | 0.74 | 0.72 | 1.61 |
| AT5G03270 | LONELY GUY 6 (*LOG6*) | 0.44 | 1.54 | 1.87 | 0.37 | 0.50 | 0.95 |
